# Stage-Specific Role of *Amelx* Activation in Stepwise Ameloblast Induction from Mouse Induced Pluripotent Stem Cells

**DOI:** 10.3390/ijms22137195

**Published:** 2021-07-03

**Authors:** Xinchao Miao, Kunimichi Niibe, Maolin Zhang, Zeni Liu, Praphawi Nattasit, Yumi Ohori-Morita, Takashi Nakamura, Xinquan Jiang, Hiroshi Egusa

**Affiliations:** 1 Division of Molecular and Regenerative Prosthodontics, Tohoku University Graduate School of Dentistry, Sendai 980-8575, Miyagi, Japan; miao.xinchao.r8@dc.tohoku.ac.jp (X.M.); zml0312@163.com (M.Z.); lznjenny0310@gmail.com (Z.L.); praphawi.nattasit.t3@dc.tohoku.ac.jp (P.N.); yumi.ohori.d4@tohoku.ac.jp (Y.O.-M.); 2 Department of Prosthodontics, Shanghai Engineering Research Center of Advanced Dental Technology and Materials, Shanghai Key Laboratory of Stomatology & Shanghai Research Institute of Stomatology, National Clinical Research Center for Oral Diseases, Shanghai Ninth People’s Hospital, College of Stomatology, Shanghai Jiao Tong University School of Medicine, Shanghai 200011, China; xinquanjiang@aliyun.com; 3 The State Key Laboratory Breeding Base of Basic Science of Stomatology (Hubei-MOST) & Key Laboratory of Oral Biomedicine Ministry of Education, School and Hospital of Stomatology, Wuhan University, Wuhan 430079, China; 4 Division of Molecular Pharmacology and Cell Biophysics, Tohoku University Graduate School of Dentistry, Sendai 980-8575, Miyagi, Japan; takashi.nakamura.d2@tohoku.ac.jp; 5 Center for Advanced Stem Cell and Regenerative Research, Tohoku University Graduate School of Dentistry, Sendai 980-8575, Miyagi, Japan

**Keywords:** ameloblast, amelogenin, cell adhesion, cell differentiation, induced pluripotent stem cells, transcriptional activation

## Abstract

Amelogenin comprises ~90% of enamel proteins; however, the involvement of *Amelx* transcriptional activation in regulating ameloblast differentiation from induced pluripotent stem cells (iPSCs) remains unknown. In this study, we generated doxycycline-inducible *Amelx*-expressing mouse iPSCs (Amelx-iPSCs). We then established a three-stage ameloblast induction strategy from Amelx-iPSCs, including induction of surface ectoderm (stage 1), dental epithelial cells (DECs; stage 2), and ameloblast lineage (stage 3) in sequence, by manipulating several signaling molecules. We found that adjunctive use of lithium chloride (LiCl) in addition to bone morphogenetic protein 4 and retinoic acid promoted concentration-dependent differentiation of DECs. The resulting cells had a cobblestone appearance and keratin14 positivity. Attenuation of LiCl at stage 3 together with transforming growth factor β1 and epidermal growth factor resulted in an ameloblast lineage with elongated cell morphology, positivity for ameloblast markers, and calcium deposition. Although stage-specific activation of *Amelx* did not produce noticeable phenotypic changes in ameloblast differentiation, *Amelx* activation at stage 3 significantly enhanced cell adhesion as well as decreased proliferation and migration. These results suggest that the combination of inducible *Amelx* transcription and stage-specific ameloblast induction for iPSCs represents a powerful tool to highlight underlying mechanisms in ameloblast differentiation and function in association with *Amelx* expression.

## 1. Introduction

Stem cell-based regenerative dentistry, with the ultimate goal of tooth regeneration, has attracted much attention in the dental field [1,2]. Tooth formation results from inductive interactions between dental epithelial cells (DECs) and mesenchymal cells [3]. DECs produce enamel, whereas dental mesenchymal cells give rise to dentin/pulp complex and periodontal tissues. Dental mesenchymal cells are retained in the dental pulp and periodontal ligament, whereas DECs, such as ameloblasts, are lost after tooth eruption, making enamel and tooth regeneration difficult [4]. Therefore, identification of alternative sources of DECs is necessary.

Induced pluripotent stem cells (iPSCs) are reprogrammed from somatic cells and show nearly unlimited self-renewal and pluripotency [5]. iPSCs have been differentiated into multiple cell lineages, including epidermal cells, chondrocytes, and osteocytes, by manipulating signaling pathways associated with in vivo development [6,7,8]. Moreover, mouse iPSCs can be differentiated into ameloblasts by ameloblast-conditioned medium or co-culture with DECs [9,10,11]. However, the factors that regulate ameloblast differentiation remain unknown.

DECs are derived from the surface ectoderm and give rise to inner enamel epithelial cells (IEEs) that differentiate into ameloblasts [3]. Ameloblast differentiation is regulated by multiple signaling pathways, such as the Wnt/β-catenin pathway. Epithelial inhibition of Wnt/β-catenin activity arrests tooth development at the lamina-early bud stage [12], whereas forced epithelial expression of β-catenin leads to continuous formation of ectopic teeth with well-differentiated ameloblasts [13]. These findings provide important clues for DEC induction in vitro. A stepwise induction strategy using signaling molecules (e.g., those related to the Wnt/β-catenin pathway) for ameloblast induction from iPSCs is under development and will contribute to developmental research in the dental field.

Genetic modification provides another strategy for directed differentiation of stem cells [14]. Controlled transcriptional regulation systems allow stage-specific activation of target genes, which mimics the process of in vivo development [15,16]. Amelogenin (AMGN), encoded by *Amelx* (AMGN, X-linked), is first expressed in IEEs and becomes predominantly expressed in secretory ameloblasts. It constitutes ~90% of the enamel matrix proteins [17,18]. Mutations in *Amelx* cause amelogenesis imperfecta in both mice and humans [19,20]. However, the roles of stage-specific transcriptional activation of *Amelx* during ameloblast induction from iPSCs remain largely unknown.

We hypothesized that the combination of doxycycline (Dox)-inducible *Amelx* transcription and a stage-specific ameloblast induction protocol for iPSCs would be a powerful method to investigate novel roles of *Amelx* transcription in ameloblast differentiation. In this study, we generated a Dox-inducible *Amelx*-expressing mouse iPSC line (Amelx-iPSCs). We then established a three-stage strategy for ameloblast induction from Amelx-iPSCs, including induction of surface ectoderm (stage 1), DECs (stage 2), and ameloblast lineage (stage 3), by manipulating several signaling factors. The objective of this study was to explore the in vitro mechanisms underlying ameloblast differentiation of mouse iPSCs in association with transcriptional activation of *Amelx*.

## 2. Results

### 2.1. Generation of Mouse Amelx-iPSCs

**Figure 1:**
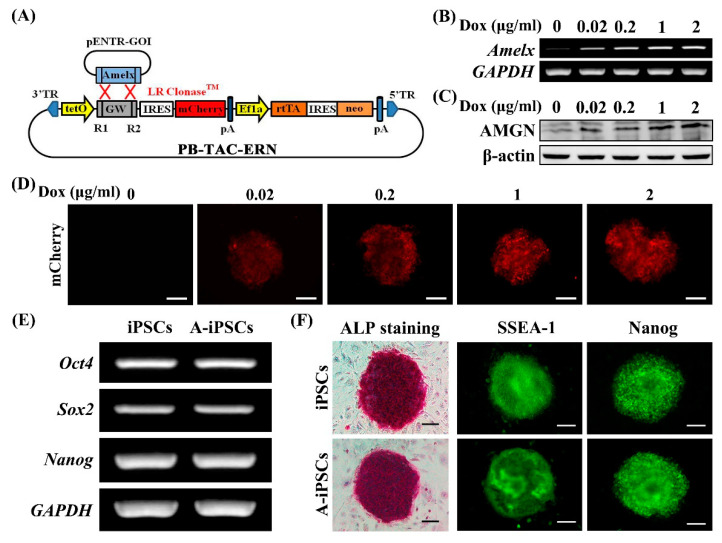
Establishment of doxycycline (Dox)-inducible *Amelx*-expressing mouse iPSC (Amelx-iPSC) line. (**A**) Generation of Dox-inducible *Amelx*-expressing *piggyBac* vector (PB-Amelx). (**B–D**) Inducible *Amelx* expression in Amelx-iPSCs after 24 h of culture with different concentrations of Dox (0–2 μg/mL) was examined by semi-quantitative RT-PCR (**B**) and Western blot (**C**) along with mCherry expression (**D**). (**E**,**F**) Amelx-iPSCs (A-iPSCs) showed pluripotency comparable to the original iPSCs, as determined by pluripotency marker gene expression (semi-quantitative RT-PCR) (**E**), ALP staining, and immunofluorescence for SSEA-1 and Nanog (**F**). Scale bars: 100 μm.

The Dox-inducible *Amelx*-expressing *piggyBac* vector with a reporter gene (mCherry) is shown in Figure 1A. Expression of *Amelx* and mCherry was induced in Amelx-iPSCs by Dox treatment at different concentrations for 24 h. We chose 1 μg/mL Dox to induce *Amelx* expression according to the observed peak expression of *Amelx* and AMGN (Figure 1B–D). Amelx-iPSCs possessed high pluripotency at nearly the same level as the original iPSCs, according to the expression of endogenous stem cell markers (*octamer-binding transcription factor 4* (*Oct4*), *SRY-box 2* (*Sox2*), and *Nanog*) by semi-quantitative reverse transcription polymerase chain reaction (RT-PCR) (Figure 1E), alkaline phosphatase (ALP) staining, and protein expression of stage-specific embryonic antigen-1 (SSEA-1) and Nanog (Figure 1F).

### 2.2. Surface Ectoderm Induction from Amelx-iPSCs by Inhibition of Nodal Signaling

iPSCs can differentiate into surface ectoderm through inhibition of Nodal signaling with SB431542 (SB43) and bone morphogenetic protein 4 (BMP4) treatment [21,22]. Therefore, we investigated the effects of SB43 and BMP4 on Amelx-iPSCs at stage 1 (Figure S1A) after embryoid body (EB) formation (Figure S1B). We found that SB43 alone showed inductive effects similar to the combination of SB43 and BMP4, resulting in abundant epithelial-like cells (Figure S1C), elevated expression of the non-neural ectoderm marker *Dlx3* and surface ectoderm marker *keratin* (*Krt*) *18*, and decreased expression of *Oct4* along with low expression of *Sox1* (neural ectoderm maker), *Brachyury* (mesoderm marker), and *Sox17* (endoderm marker) (Figure S1D). Thus, we used SB43 alone for surface ectoderm induction at stage 1 (days 2–5).

### 2.3. Concentration-Dependent Promotion of DEC Induction by Lithium Chloride (LiCl)

**Figure 2:**
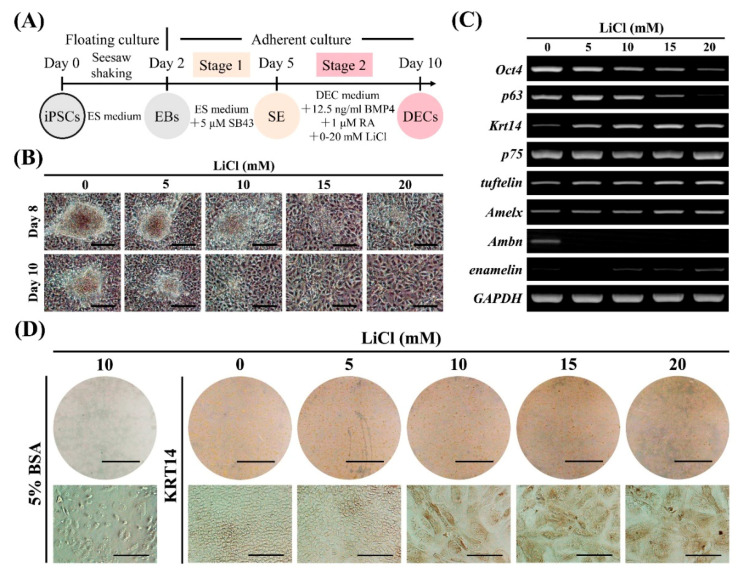
Effects of LiCl concentration on dental epithelial cell (DEC) induction. (**A**) Diagram of DEC induction from Amelx-iPSCs. After surface ectoderm (SE) induction (stage 1) by SB43 (nodal signaling inhibitor), the cells were treated with BMP4, RA, and LiCl (Wnt/β-catenin pathway activator: 0–20 mM) in DEC medium for DEC induction (stage 2). (**B**) Cell morphology at days 8 and 10 during stage 2 of induction. Scale bars: 200 μm. (**C**) Gene expression of stemness (*Oct4*), proliferative epithelium (*p63*), DEC (*Krt14*, *p75*, and *tuftelin*), and ameloblast (*Amelx*, *Ambn*, and *enamelin*) markers as determined by semi-quantitative RT-PCR analysis after stage 2 (on day 10). (**D**) Immunocytochemistry for KRT14 (dental epithelial marker) after stage 2 (on day 10). Scale bars: 1 cm and 100 μm for upper and lower panels, respectively.

Retinoic acid (RA) and BMP4 signaling synergize to induce epithelial cells from human embryonic stem cells [23,24]. Moreover, epithelial Wnt/β-catenin activity is required for tooth initiation [12]. Thus, we hypothesized that the activation of Wnt/β-catenin activity by LiCl together with RA and BMP4 could promote DEC induction at stage 2 (Figure S2A). The combination of BMP4, RA, and LiCl (10–20 mM) resulted in epithelial-like cells (Figure S2B) with decreased *Oct4* expression and increased expression of the dental epithelial markers *Krt14* and *tuftelin* (Figure S2C); however, the cells did not survive at a higher concentration of 50 mM LiCl (Figure S2B). These results suggest that LiCl promotes DEC induction in a concentration-dependent manner. Thus, we used a series of LiCl concentrations up to 20 mM to optimize the induction protocol at stage 2 (Figure 2A; days 5–10). Increased concentrations of LiCl produced gradual changes in cell morphology from a small oval shape to a large cobblestone appearance (Figure 2B), accompanied by gradual downregulation of *Oct4* and *p63*, a proliferative epithelial marker [25], and upregulation of *Krt14, tuftelin*, and *Amelx* (Figure 2C). Additionally, *p75*, which is expressed in IEEs and pre-ameloblasts [18], was highly expressed in all groups, whereas expression of the ameloblast markers *ameloblastin* (*Ambn*) and *enamelin* was weak or even undetected (Figure 2C). Immunocytochemistry revealed increased KRT14 expression at higher concentrations of LiCl, with a peak at 15 mM (Figure 2D). Thus, we chose 15 mM LiCl for stage 2 induction.

### 2.4. Ameloblast Lineage Differentiation by LiCl, Epidermal Growth Factor (EGF), and Transforming Growth Factor β1 (TGF-β1)

Epithelial deletion of β-catenin during the tooth differentiation stage leads to insufficient ameloblast differentiation and enamel formation in mice [26]. Additionally, TGF-β1 promotes ameloblast differentiation ex vivo [27] and enhances ameloblast differentiation in the presence of EGF [28]. Moreover, SF2-differentiation (SFD) medium promotes ameloblast differentiation of rat DECs [29]. Thus, we investigated SFD medium supplemented with LiCl, EGF, and TGF-β1 for ameloblast induction at stage 3 (days 10–17; Figure S3A). SFD medium alone did not significantly affect cell morphology (Figure S3B) and did not fully promote the expression of ameloblast markers (Figure S3C). In contrast, addition of LiCl (15 mM), EGF, and TGF-β1 to SFD medium promoted ameloblast lineage induction characterized by an elongated cell shape (Figure S3B), high expression of ameloblast markers (*Amelx*, *Ambn*, and *enamelin*), low expression of *Oct4* (Figure S3C), and positive staining for KRT14 and AMGN (Figure S3D).

### 2.5. Optimization of LiCl Concentrations at Stages 2 and 3 for Ameloblast Induction

**Figure 3:**
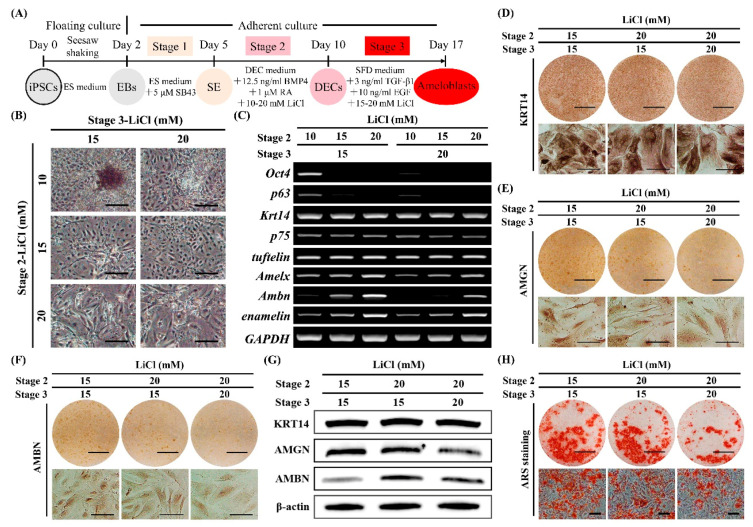
Optimization of LiCl concentrations at stages 2 and 3 for ameloblast induction. (**A**) Diagram of ameloblast induction from Amelx-iPSCs. Different concentrations of LiCl were tested at stages 2 (10, 15, and 20 mM) and 3 (15 and 20 mM) to optimize the stage-specific concentrations for ameloblast induction. (**B**) Cell morphology after stage 3 induction (on day 17). Scale bars: 200 μm. (**C**) Gene expression as determined by semi-quantitative RT-PCR analysis after stage 3 (on day 17). Marker expression (stemness: *Oct4*; proliferative epithelium: *p63*; dental epithelial cells (DECs): *Krt14*, *p75*, and *tuftelin*; and ameloblasts: *Amelx*, *Ambn*, and *enamelin*). (**D–H**) Evaluation of ameloblast phenotypes, with higher expression of ameloblast marker genes after stage 3 (on day 17), as determined by immunocytochemistry for KRT14 (**D**), AMGN (**E**), and AMBN (**F**); Western blot (**G**); and Alizarin Red S (ARS) staining (**H**). Scale bars: 1 cm and 100 μm for upper and lower panels, respectively.

Because LiCl promoted DEC differentiation in a concentration-dependent manner at stage 2, we hypothesized that different combinations of LiCl concentrations during stages 2 and 3 would affect ameloblast differentiation. We applied three conditions (10, 15, and 20 mM LiCl) at stage 2 and two conditions (15 and 20 mM LiCl) at stage 3 (Figure 3A). Cells treated with 20 mM LiCl at stage 2 and 15 mM LiCl at stage 3 (stage 2/stage 3: 20/15 mM) showed elongated spindle-like shapes (Figure 3B) that resembled polarized ameloblasts and the highest expression of *Amelx*, *Ambn*, and *enamelin*; attenuated *Oct4* and *p63* expression; and unaffected expression of *Krt14*, *p75*, and *tuftelin* (Figure 3C). Most cells in each LiCl concentration condition that showed higher expression of ameloblast markers as determined by RT-PCR analysis (stage 2/stage 3: 15/15 mM, 20/15 mM, and 20/20 mM) also showed positive staining for KRT14, AMGN, and AMBN (Figure 3D–F), with higher average AMGN and AMBN expression with 20 mM LiCl at stage 2 and 15 mM LiCl at stage 3. This confirmed the optimal combination of LiCl concentrations (Figure 3G). Additionally, Alizarin Red S (ARS) staining showed comparable results between the 15/15 mM and 20/15 mM conditions and decreased staining intensity in the 20/20 mM group (Figure 3H). These results suggest that attenuation of Wnt/β-catenin activity at stage 3 following stage 2 was beneficial for ameloblast induction.

Using the established protocol (Figure S4A), the original mouse iPSCs showed ameloblast induction comparable to that of mouse Amelx-iPSCs, as determined by the gene expression of ameloblast markers (Figure S4B) and protein levels of KRT14, AMBN, and AMGN (Figure S4C,D), suggesting that this protocol would also be suitable for native mouse iPSCs, even without forced *Amelx* expression.

### 2.6. Gene Expression Profile of Ameloblast Differentiation Markers during the Stepwise Induction of Amelx-iPSCs

**Figure 4:**
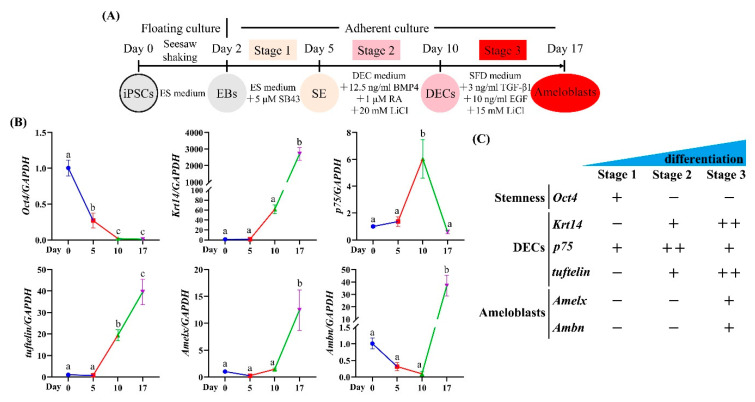
Gene expression profile of ameloblast differentiation markers during the stepwise induction of Amelx-iPSCs. (**A**) Diagram of an optimized protocol for stepwise ameloblast induction from mouse iPSCs. (**B**) Gene expression of stemness (*Oct4*) and ameloblast differentiation (dental epithelial cells (DECs): *Krt14*, *p75*, and *tuftelin*; and ameloblasts: *Amelx* and *Ambn*)) markers at each stage determined by real-time RT-PCR during stepwise induction of ameloblast lineage from Amelx-iPSCs. Different letters among groups (e.g., a, b) indicated significant differences (*p* < 0.05; one-way ANOVA and Tukey’s test; *n* = 3). (**C**) Summary of gene expression profile of ameloblast differentiation markers during the stepwise induction of mouse iPSCs.

After optimization of the stepwise ameloblast induction protocol (Figure 4A), we evaluated gene expression patterns at individual induction stages. *Oct4* expression gradually decreased as ameloblast induction advanced and was almost undetectable after stage 3 (Figure 4B). The expression of *Krt14* and *tuftelin* was first upregulated at stage 2 and significantly increased at stage 3 (*p* < 0.05; one-way ANOVA and Tukey’s test) (Figure 4B). *p75* expression reached a peak at stage 2 but markedly decreased at stage 3, whereas *Amelx* and *Ambn* expression were low at stage 2 but significantly increased at stage 3 (*p* < 0.05; one-way ANOVA and Tukey’s test) (Figure 4B). These results show a clear stage-specific profile of differential gene expression (Figure 4C) and suggest that our 3-stage induction protocol might mimic the in vivo developmental process.

### 2.7. Effects of Stage-Specific Transcriptional Activation of Amelx during Stepwise Ameloblast Induction

**Figure 5:**
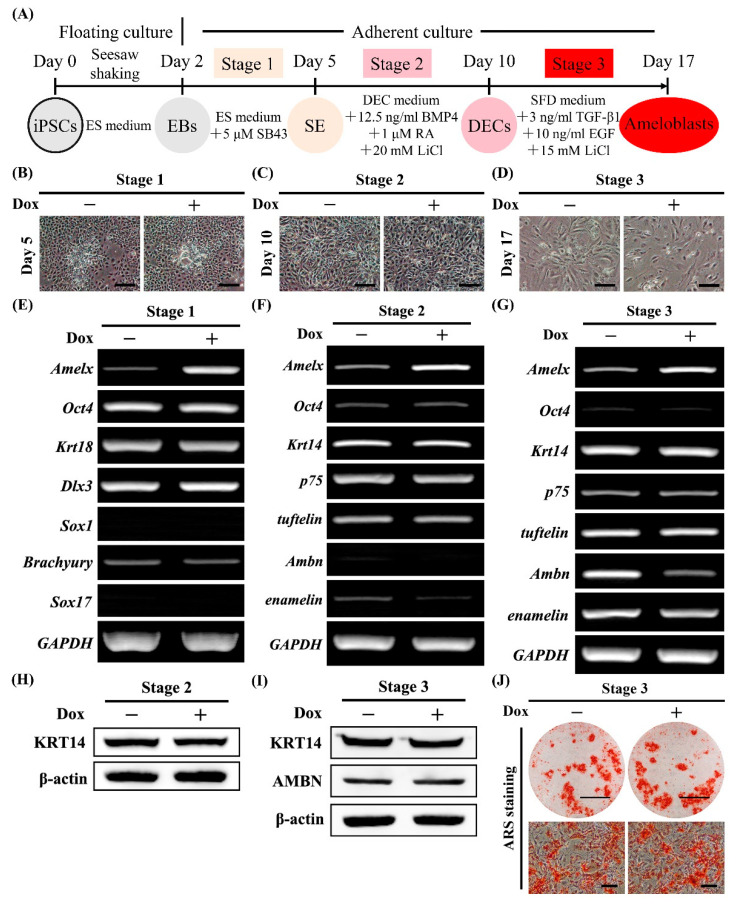
Effects of stage-specific transcriptional activation of *Amelx* on ameloblast differentiation of Amelx-iPSCs. (**A**) Diagram of the established stepwise induction protocol. (**B**–**D**) Cell morphology of Amelx-iPSCs on day 5 (**B**), 10 (**C**), and 17 (**D**) with or without Dox treatment during stage 1 (**B**), stage 2 (**C**) or stage 3 (**D**). Scale bars: 200 μm. (**E**–**G**) Gene expression of stemness (*Oct4*), surface ectoderm (*Krt18*), non-neural ectoderm (*Dlx3*), neural ectoderm (*Sox1*), mesoderm (*Brachyury*), endoderm (*Sox17*) DEC (*Krt14*, *p75*, and *tuftelin*), and ameloblast (*Amelx*, *Ambn*, and *enamelin*) markers in Amelx-iPSCs on day 5 (**E**), 10 (**F**), and 17 (**G**) with or without Dox treatment during stage 1 (**E**), stage 2 (**F**), or stage 3 (**G**) as determined by semi-quantitative RT-PCR analysis. (**H-I**) Expression of KRT14 and AMBN in Amelx-iPSCs on day 10 (**H**) and 17 (**I**) with or without Dox treatment during stage 2 (**H**) or stage 3 (**I**) as determined by Western blotting. β-actin was used as an internal control. (**J**) Alizarin Red S (ARS) staining on day 17 for Amelx-iPSCs with or without Dox treatment during stage 3. Scale bars: 1 cm and 100 μm for upper and lower panels, respectively.

We evaluated the effects of stage-specific transcriptional activation of *Amelx* on ameloblast differentiation of Amelx-iPSCs by addition of Dox (Figure 5A). *Amelx* activation during each stage did not significantly affect cell morphology (Figure 5B–D) or expression of ameloblast differentiation-associated molecules at the respective stage (Figure 5E–I). *Amelx* activation during stage 3 downregulated *Ambn* transcripts (Figure 5G), but did not substantially affect expression of AMBN protein (Figure 5I). In addition, ARS staining on day 17 was similar between Amelx-iPSCs with or without Dox treatment during stage 3 (Figure 5J), suggesting that *Amelx* activation did not robustly affect ameloblast differentiation.

**Figure 6:**
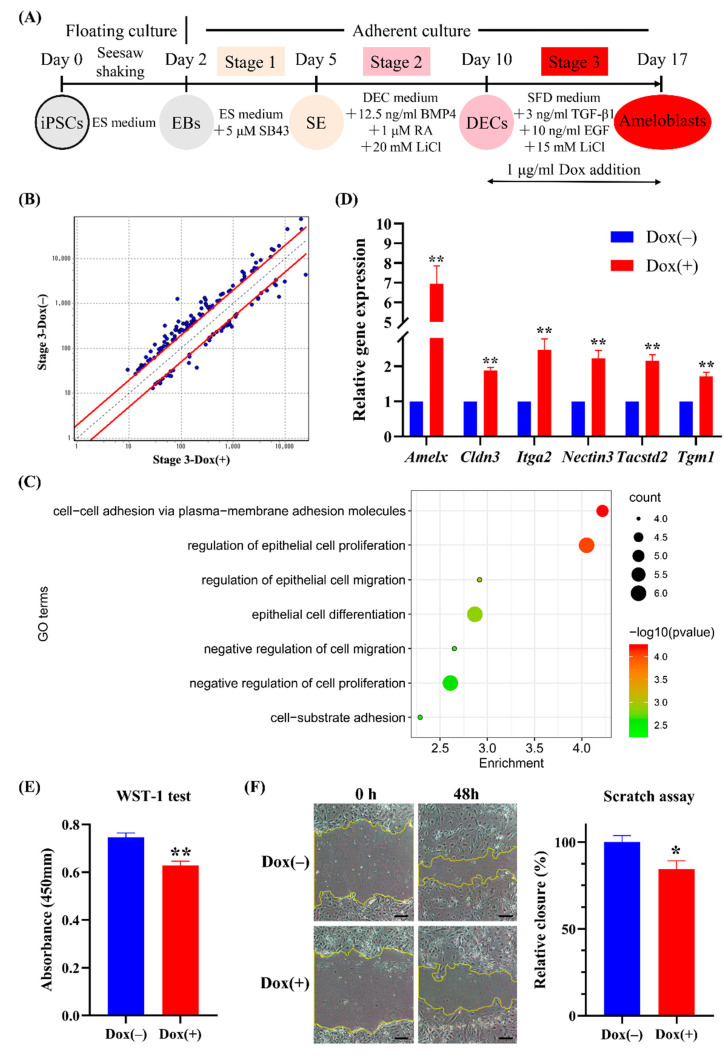
Effects of transcriptional activation of *Amelx* in Amelx-iPSCs at stage 3 by microarray analysis. (**A**) Diagram of induction protocol. Using the established stepwise ameloblast induction from Amelx-iPSCs, 1 μg/mL doxycycline (Dox) was added to the induction medium during stage 3, and microarray analysis was performed after stage 3 (on day 17). (**B**) Scatter diagram of differentially expressed genes (DEGs) identified by microarray analysis. (**C**) Gene ontology (GO) terms associated with the upregulated DEGs. (**D**) Verification of six upregulated DEGs (*Amelx*, *Cldn3*, *Itga2*, *Nectin3*, *Tacstd2*, and *Tgm1*) by real-time RT-PCR. ** *p* < 0.01 (*t*-test; *n* = 3). (**E**) Cell proliferation measured by the WST-1 assay on day 17. ** *p* < 0.01 (*t*-test; *n* = 3). (**F**) Representative images and quantification of scratch assay for evaluation of cell migration. Scratch assay was performed on day 13 at stage 3. The cell migration was measured at 0 and 48 h after cell scraping. Scale bars: 200 μm. * *p* < 0.05 (*t*-test; *n* = 3).

Because *Amelx* was predominantly expressed in ameloblast lineages corresponding to stage 3 of our induction protocol (Figure 4B), we used whole-transcript expression arrays to identify differentially expressed genes (DEGs) upon transcriptional activation of *Amelx* during stage 3 (Figure 6A). Microarray analysis identified 46 upregulated DEGs and 106 downregulated DEGs in Dox-treated cells (Figure 6B). Moreover, gene ontology (GO) analysis [30] revealed that the upregulated DEGs were associated with adhesion, proliferation, migration, and differentiation of epithelial cells (Figure 6C and Table S1), whereas the downregulated DEGs were not associated with epithelial cell-related processes (Figure S5 and Table S2). Real-time PCR analysis revealed that the six candidate genes among the upregulated DEGs (*Amelx*, *claudin3* (*Cldn3)*, *nectin cell-adhesion molecule 3* (*Nectin3*), *integrin α2* (*Itga2*), *tumor-associated calcium signal transducer 2* (*Tacstd2*), and *transglutaminase 1, K polypeptide* (*Tgm1*)) had significantly higher expression in the Dox(+) group (*p* < 0.01; *t*-test) (Figure 6D), which agreed with the microarray data. We also determined the cell proliferation and migration after Dox treatment at stage 3 by WST-1 and scratch assays, respectively. The results showed that Dox treatment at stage 3 significantly decreased cell proliferation (*p* < 0.01; *t*-test) (Figure 6E) and migration (*p* < 0.05; *t*-test) (Figure 6F), indicating that *Amelx* activation in ameloblast lineage negatively regulated cell proliferation and migration.

**Figure 7:**
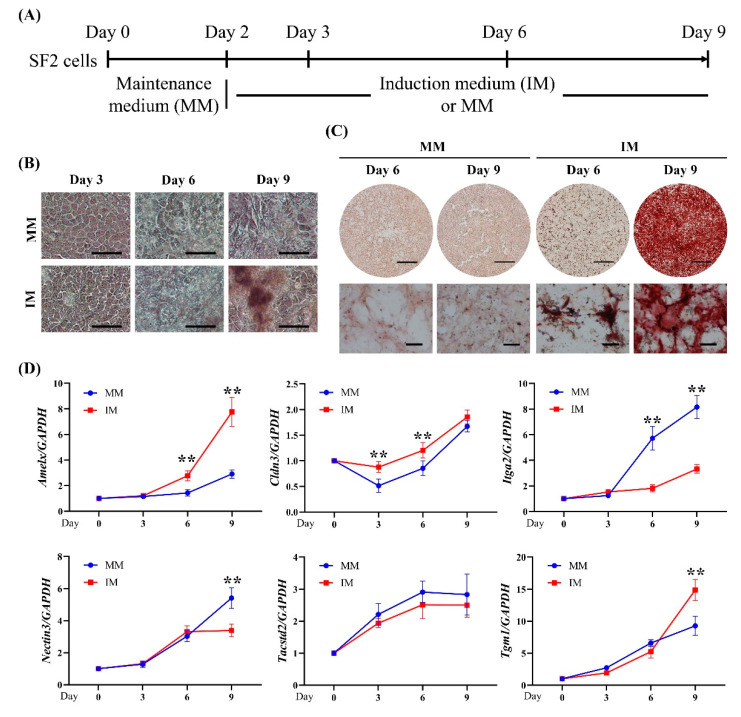
Verification of the upregulated DEGs in a rat dental epithelial cell line (SF2 cells). (**A**) Diagram of SF2 cell induction toward ameloblasts. (**B**) Cell morphology at days 3, 6, and 9. Scale bar: 100 μm. (**C**) Alizarin Red S staining at days 6 and 9. Scale bars: 5 mm and 200 μm for upper and lower panels, respectively. (**D**) Expression pattern of the upregulated DEGs during ameloblast differentiation of SF2 cells as determined by real-time RT-PCR analysis. Significant differences (** *p* < 0.01: two-way ANOVA with Sidak’s test; *n* = 3) were evaluated with respect to the values between MM and IM at each time point.

We subsequently determined the expression pattern of these genes during ameloblast induction of SF2 cells, a DEC line (Figure 7A). Phase-contrast microscopy revealed that extracellular matrix content increased over time in both conditions (Figure 7B); however, ARS-stained nodules were only abundant in cells treated with ameloblast induction medium. Few nodules were observed in cells incubated in maintenance medium (Figure 7C). Additionally, *Amelx* expression increased over time in cells treated with induction medium as compared to cells incubated in maintenance medium (*p* < 0.01; two-way ANOVA with Sidak’s test) (Figure 7D). The slight upregulation of *Amelx* at day 9 in the cells incubated in the maintenance medium might indicate spontaneous ameloblast differentiation. Elevated expression of *Cldn3*, *Itga2*, *Nectin3*, *Tacstd2*, and *Tgm1* in SF2 cells during ameloblast differentiation was confirmed over time for cells in both the induction and maintenance media (Figure 7D).

## 3. Discussion

In this study, we successfully generated Dox-inducible *Amelx*-expressing mouse Amelx-iPSCs, established a 3-stage strategy for ameloblast induction from Amelx-iPSCs, and determined the effect of stage-specific transcriptional activation of *Amelx* during ameloblast induction following our protocol. Our stepwise ameloblast induction protocol not only allows mass production of ameloblast lineages by defined factors used for regenerative dentistry, but also provides an in vitro platform for stage-specific determination of target genes associated with ameloblast differentiation.

Ameloblast differentiation is regulated by multiple signaling pathways and involves hundreds of molecules identified by in vivo studies, such as Wnt/β-catenin, BMP4, and TGF-β1 [3] (details in https://bite-it.helsinki.fi/, accessed on 15 June 2021). Mimicking such a complex regulatory network for in vitro ameloblast induction with a specific combination of known signaling molecules remains challenging. Previous studies show that ameloblast lineage could be induced from iPSCs by co-culture with rat DECs [9], or conditional medium from epithelial cell rests of Malassez [10] or ameloblasts [11]. However, in these reports, the components that regulate ameloblast differentiation remain unknown. In the present study, we developed a three-stage ameloblast induction strategy by manipulating signaling pathways associated with in vivo development. Our induction protocol is based on defined molecules, avoiding interfering effects from unknown factors released by epithelial cells on iPSC-induced ameloblasts. To our best knowledge, this is the first study to generate ameloblast lineage cells from iPSCs by defined signaling factors, which allows mass production of ameloblast lineage cells.

It is well known that Nodal signaling is required for mesendoderm differentiation, and inhibition of Nodal signaling promotes ectoderm development from pluripotent stem cells [21,31]. Ectoderm cells differentiate spontaneously into neural ectoderm, which could be blocked by BMP4, and then the cells adopt a surface ectoderm fate [21]. In our protocol, SB43 alone showed inductive effects similar to the combination use of SB43 and BMP4. Moreover, spontaneous neural induction was blocked in the control group (i.e., ES medium), and the addition of BMP4 to ES medium showed results similar to ES medium alone, suggesting that endogenous BMP signaling might be activated in the ES medium. Therefore, only SB43 treatment could promote SE induction from mouse iPSCs in the ES medium by endogenous activation of BMP signaling.

Among multiple signaling pathways, Wnt/β-catenin signaling was thought to be the single most important pathway for initiation of tooth development [13,32,33]. In the present study, we identified that LiCl, a Wnt/β-catenin pathway activator, together with BMP4 and RA promoted DEC induction at stage 2 in a concentration-dependent manner (Figure 2). *Krt14* is a member of the type 1 keratin family, which is expressed in the stratified epithelial cells, including DEGs, IEEs, and ameloblasts [18,34]. Besides, *tuftelin*, an acid-phosphorylated glycoprotein, was initially expressed at early DECs and continuously expressed in IEEs and ameloblasts [17]. After stage 2 induction, iPSC-derived cells expressed these dental epithelial markers in a dose-dependent manner, indicating that our induction protocol promoted DEC differentiation from iPSCs. Low concentrations of LiCl below 5 mM showed minimal effects, whereas higher concentrations of LiCl at 10–20 mM promoted DEC induction indicated by upregulation of DEC markers. These results suggest that the Wnt/β-catenin pathway could also initiate DEC differentiation from iPSCs in vitro, in line with the in vivo results.

Moreover, in a previous study, epithelial deletion of β-catenin during the tooth differentiation stage resulted in insufficient ameloblast differentiation as shown by decreased *Amelx* expression [26], suggesting that Wnt/β-catenin activity is also required during ameloblast differentiation. We found that LiCl treatment upregulated *Amelx* but downregulated *Ambn* at stage 3, whereas a combination of LiCl, TGF-β1, and EGF enhanced ameloblast induction as revealed by higher expression of *Amelx*, *Ambn,* and *enamelin* and positivity for KRT14 and AMGN (Figure S3). Furthermore, we found that attenuation of LiCl (15 mM) at stage 3 following administration of 20 mM LiCl at stage 2 resulted in the highest expression of ameloblast markers (KRT14, AMGN, and AMBN), suggesting subtle regulation of the Wnt/β-catenin pathway during ameloblast induction (Figure 3). Following the stepwise induction, mouse iPSCs were guided toward an ameloblast lineage characterized by elongated cell morphology and positive staining for ameloblast markers and calcium deposition. We observed distinct stage-specific differences in cell morphology and gene expression patterns (Figure 2, Figure 3 and Figure 4), similar to patterns present during in vivo development. Collectively, these results expand the current understanding of regulatory molecules during the stages of ameloblast differentiation, addressing the importance of manipulation of the Wnt/β-catenin pathway for ameloblast lineage induction.

It is known that *Amelx* is required for enamel formation [19,20]. Because *Amelx* is predominantly expressed in ameloblast lineages [17,18], we asked whether *Amelx* activation could promote ameloblast induction from iPSCs. By using a tetracycline-controlled transcriptional regulation system, we established Dox-inducible Amelx-iPSCs, which allowed us to activate *Amelx* expression at the desired stage to mimic the expression pattern in vivo. However, we found that stage-specific activation of *Amelx* did not dramatically affect ameloblast differentiation (Figure 5). Nonetheless, *Amelx* overexpression during the ameloblast induction stage was associated with enhanced cell adhesion as well as negative proliferation and migration (Figure 6). *Nectin3* and *Tgm1* are involved in the formation of adherens junctions localized between ameloblasts and the stratum intermedium [35,36]. *Cldn3* and *Tacstd2* participate in the formation of tight junctions between ameloblasts [37,38]. *Itga2* mediates adhesion between ameloblasts and the basement membrane [39]. Other roles of *Cldn3* and *Tacstd2*, such as negative regulation of the proliferation and migration of epithelial cells, have also been reported [40,41]. In the present study, we found that all of these genes were upregulated by transcriptional activation of *Amelx* during stage 3. Furthermore, we confirmed the physiological upregulation of these genes in a DEC line during ameloblast differentiation. Our results suggest for the first time the involvement of these genes in ameloblast induction in association with *Amelx* expression. Despite the obvious differences between the maintenance and induction media in terms of extracellular matrix mineralization by SF2 cells, both culture conditions produced upregulation of *Cldn3*, *Nectin3*, *Tacstd2*, and *Tgm1*, suggesting that these genes are not specifically associated with ameloblast mineralization. It seems that transcriptional activation of *Amelx* in iPSCs does not directly affect ameloblast differentiation, but rather stimulates gene sets associated with cell proliferation and adhesion during ameloblast induction, which agrees with a previous report showing that amelogenin regulates the proliferation and adhesion of periodontal ligament cells [42]. Taken together, these results imply that *Amelx* activation might enhance the ameloblast-stratum intermedium, ameloblast-ameloblast, and ameloblast-basement membrane interactions, while inhibiting cell proliferation and migration during ameloblast development. Further in vivo research in *Amelx* knock-in mice is needed to confirm this mechanism.

Our results suggest that the combination of inducible transcription of interested genes, such as *Amelx*, and a stage-specific ameloblast induction protocol for iPSCs represents a powerful tool for biomedical discoveries. Such a platform could help to uncover new functions of target genes and new functional molecules during ameloblast development. Further research is needed to determine the mechanisms by which the multiple factors tested regulate DEC differentiation during stages 2 and 3 of our protocol. Additionally, it will be of interest to investigate the in vivo behavior of induced ameloblast lineages, including their efficacy when combined with dental mesenchyme to generate tooth-like organs (i.e., bioengineered teeth).

In conclusion, we developed a three-stage ameloblast induction strategy using a stage-specific combination of several signaling molecules. Using this induction protocol, we found that transcriptional activation of *Amelx* during ameloblast induction at the late stage enhanced cell adhesion and decreased proliferation and migration. This work expands the current understanding of regulatory networks during ameloblast differentiation and provides an in vitro platform with practical impact for stage-specific evaluation of genes involved in ameloblast induction.

## 4. Materials and Methods

### 4.1. Establishment of Mouse Amelx-iPSCs

This project was approved by the Center and Committee of Gene Research, Tohoku University (approval nos. 2015DnLMO-008, 2017DnLMO-011, and 2020DnLMO-007). The pENTR 221 Gateway Entry vector (Thermo Fisher Scientific, Waltham, MA, USA) containing full-length cDNA for mouse *Amelx* (GenBANK: BC059090.1) [43] was used to generate the *Amelx*-expressing vector. Mouse gingival fibroblast-derived iPSCs were propagated in ES medium as previously described [44]. The establishment of mouse Amelx-iPSCs followed a previously described protocol using the *piggyBac* transposon system [15]. The details are described in the Supplementary Materials.

### 4.2. Stepwise Induction of Mouse iPSCs into Ameloblast Lineage

Mouse Amelx-iPSCs or the original mouse iPSCs were dissociated to single cells by trypsin-EDTA, followed by addition of 3.0 × 10^5^ iPSCs/well to low-attachment 6-well plates (Thermo Fisher Scientific) in ES medium and cultured for 2 days under seesaw shaking conditions (speed: 30 rpm; angle: 8° using a shaker (Taitec Corporation, Koshigaya, Japan) for EB formation. The EBs were collected by centrifugation, seeded onto gelatin-coated plates (Greiner Bio-One, Kremsmunster, Austria), and subjected to a stepwise-induction protocol, including induction of surface ectoderm, DECs, and ameloblast lineage in sequence.

For surface ectoderm induction (stage 1), EBs were cultured in ES medium with or without 5 μM SB43 (nodal signaling inhibitor; Sigma-Aldrich, St. Louis, MO, USA) or 35 ng/mL BMP4 (Peprotech, Rocky Hill, NJ, USA) for 3 days [21,22]. The medium was refreshed every other day. Surface ectoderm differentiation at stage 1 was examined by cell morphology and semi-quantitative RT-PCR (*n* = 2). The primer sequences are shown in Table S3. Experiments were repeated at least 2 times with similar results.

Cells were then cultured in DEC medium (Dulbecco’s modified Eagle medium (DMEM)/F12 (Thermo Fisher Scientific), 20 ng/mL EGF (Wako, Osaka, Japan), 25 ng/mL basic fibroblast growth factor (Wako), 1× B27 supplement (Thermo Fisher Scientific), and 1% penicillin/streptomycin [45]) supplemented with 12.5 ng/mL BMP4, 1 μM all-trans RA (Wako), and up to 50 mM LiCl (Wako) with daily medium change for 5 days to guide DEC differentiation (stage 2). DEC differentiation at stage 2 was evaluated by cell morphology, semi-quantitative RT-PCR (*n* = 2), and immunocytochemistry (*n* = 2). Experiments were repeated at least 2 times with similar results.

Cells were then induced to ameloblast lineage (stage 3) using SFD medium supplemented with 10 ng/mL EGF, 3 ng/mL TGF-β1 (Peprotech), and 15 mM to 20 mM LiCl for 7 days, with the medium changed every other day. The SFD medium contained α-MEM (Nacalai Tesque, Kyoto, Tokyo) with 10% fetal bovine serum (FBS; Thermo Fisher Scientific), 20 mM β-glycerophosphate (Thermo Fisher Scientific), 50 μg/mL ascorbic acid (Thermo Fisher Scientific), 0.1 μM calcitriol (Wako), 2 mM calcium chloride (Wako), and 1% penicillin/streptomycin, which was modified from a previous study [29]. Ameloblast differentiation at stage 3 was analyzed by cell morphology, semi-quantitative RT-PCR (*n* = 2), Western blotting (*n* = 2), immunocytochemistry (*n* = 2) and ARS staining (*n* = 2). Experiments were repeated at least 2 times with similar results. The details for each experiment are described in the Supplementary Materials.

### 4.3. Stage-Specific Transcriptional Activation of Amelx during Stepwise Induction of Ameloblast Lineage

To determine the stage-specific roles of transcriptional activation of *Amelx* during induction, Dox (1 μg/mL) was added to the induction medium [43] at the indicated stages according to the established induction protocol. We evaluated cell morphology and performed semi-quantitative RT-PCR (*n* = 2), Western blot analyses (*n* = 2), and ARS staining (*n* = 2). Additionally, whole-transcript expression microarrays were performed using total RNA samples (*n* = 1) on day 17 (end point of stage 3); real-time RT-PCR (*n* = 3), WST-1 (*n* = 3), and scratch assays (*n* = 3) were used to verify the results of the microarray analysis. Experiments except the microarray analysis were repeated at least 2 times, and similar results were obtained. The details for each experiment are described in the Supplementary Materials.

### 4.4. SF2 Induction toward Ameloblasts

The rat DEC line SF2 was maintained in DMEM/F12 (Thermo Fisher Scientific) with 10% FBS (Thermo Fisher Scientific) and 1% penicillin/streptomycin (maintenance medium) [29]. SF2 cells (3.0 × 10^5^ cells/well) were seeded onto gelatin-coated 6-well plates in maintenance medium for 2 days, after which the cells were incubated in SFD medium as the induction medium for 7 days, with maintenance medium used as a control [29]. Cell morphology, real-time RT-PCR (*n* = 3), and ARS staining (*n* = 2) were conducted to investigate SF2 cell differentiation toward ameloblasts. Experiments were repeated at least 2 times with similar results.

### 4.5. Experimental Protocols

The detailed experimental protocols are provided in the Supplementary Materials.

### 4.6. Statistical Analysis

For quantitative data, results were expressed as the mean ± standard deviation (*n* = 3). Statistically significant differences (*p* < 0.05) were identified by unpaired t test, one-way ANOVA (Tukey’s test), or two-way ANOVA (Sidak’s test) using the GraphPad Prism statistical software package (version 8.0; GraphPad Software Inc., San Diego, CA, USA).

## Data Availability

Not applicable.

## Supplementary Materials

The following are available online at https://www.mdpi.com/article/10.3390/ijms22137195/s1 [15,30,44] are cited in supplementary materials. Figure S1: Surface ectoderm (SE) induction (stage 1) from Amelx-iPSCs by inhibition of Nodal signaling, Figure S2: Combination of LiCl, retinoic acid (RA), and BMP4 promoted dental epithelial cell (DEC) induction, Figure S3: LiCl, EGF, and TGF-β1 cooperated to induce ameloblast lineage differentiation, Figure S4: Application of the established stepwise induction protocol to the original mouse iPSCs, Figure S5: Gene ontology (GO) terms for the downregulated DEGs in the microarray analysis, Table S1: GO enrichment analysis of upregulated genes, Table S2: GO enrichment analysis of downregulated genes, Table S3: Primers used for semi-quantitative RT-PCR and real-time RT-PCR.
