# Supplementary material for: Stage-Specific Role of Amelx Activation in Stepwise Ameloblast Induction from Mouse Induced Pluripotent Stem Cells
Source: Int J Mol Sci. 2021 Jul 3;22(13):7195. doi: 10.3390/ijms22137195 (PMC8268366; doi:10.3390/ijms22137195)
Supplement: Supplementary file 1 [file ijms-22-07195-s001.zip › ijms-1275887-SI.pdf]

Stage-Specific Role of *Amelx* Activation in Stepwise Ameloblast Induction from Mouse Induced Pluripotent Stem Cells

Supplementary Materials and Methods

Mouse iPSC Culture

Mouse gingival fibroblast-derived iPSCs were propagated in ES medium as previously described [1]. Briefly, mouse iPSCs were cultured on inactivated SNLP76.7-4 feeder cells in ES medium: DMEM (Nacalai Tesque, Kyoto, Japan) with 15% FBS (Thermo Fisher Scientific, Waltham, MA, USA), 2 mM L-glutamine (Wako, Osaka, Japan), 1×10^{-4} M nonessential amino acids (Thermo Fisher Scientific), 1×10^{-4} M 2-mercaptoethanol (Thermo Fisher Scientific), and 0.5% penicillin/streptomycin (Wako). Mouse iPSCs were passaged every 5–6 days using trypsin-EDTA (Wako).

Establishment of Doxycycline-Inducible Amelx-Expressing Mouse iPSC Line (Amelx-iPSCs).

This project was approved by the Center and Committee of Gene Research, Tohoku University. The pENTR 221 Gateway Entry vector containing full-length cDNA of mouse *Amelx* (GenBANK: BC059090.1) was purchased from Thermo Fisher Scientific. The PB-TAC-ERN (KW111) vector (All-in-One piggyBac transposon destination vector) and pCAG-PBase expression vector (KW158) were kindly provided by Dr. Knut Woltjen (Kyoto University, Japan) [2]. The *Amelx* cDNA was transferred to the PB-TAC-ERN vector to generate the transposon PB-Amelx (Fig.1A) by the LR reaction.

Dox-inducible mouse Amelx-iPSCs were obtained via the Neon transfection system (Thermo Fisher Scientific) following the manufacturer’s instructions. In brief, 1 µg of destination vector (PB-Amelx) and 1 µg of PBase plasmid (KW158) were mixed with 100 µL of mouse iPSC suspension (1.0 $\times 10^7$ cells/mL), and then the cell-DNA mixture was subjected to electroporation treatment with the given parameters (pulse voltage: 1300 v; pulse width: 20 ms; pulse no.: 2). At 48 h after transfection, 150 mg/mL G418 (Wako) was added to select positive Amelx-iPSC clones. After 5 days of G418 treatment, clones were picked, and appropriate mouse Amelx-iPSC clones with high mCherry expression in the presence of 1 µg/mL Dox (Sigma-Aldrich, St Louis, MO, USA) were selected for use.

The Dox-inducible gene expression system was shown to be concentration-responsive in a previous report [2]. To optimize the concentration of Dox, four different concentrations of Dox (0.02, 0.2, 1, and 2 µg/mL) were added to the culture medium, and the expression of *Amelx* was examined using mCherry expression, reverse transcription polymerase chain reaction (RT-PCR), and western blotting after 24 h.

The pluripotency of mouse Amelx-iPSCs was evaluated by comparing alkaline phosphatase (ALP) staining, immunofluorescence staining for Nanog and SSEA-1, and RT-PCR analysis of

endogenous *Sox2*, *Oct4*, and *Nanog* relative to the original mouse iPSCs.

ALP Staining

Cells were washed with PBS, fixed with 10% neutral buffered formalin, and stained with 120 mM Tris buffer (pH 8.4) containing 1.8 mM fast red TR (Sigma-Aldrich) and 0.9 mM naphthol AS-MX phosphate (Sigma-Aldrich) for 30 min at 37 °C.

ARS Staining

After washing with PBS and fixation with 10% neutral buffered formalin, cells were incubated with 40 mM ARS (pH 4.1) for 20 min under gentle shaking. After washing four times with distilled water, the samples were scanned to generate digital images.

Semi-Quantitative RT-PCR and Real-Time RT-PCR

Total RNA was extracted using TRIzol (Thermo Fisher Scientific) and purified using DNase I treatment (Thermo Fisher Scientific). First-strand cDNA was synthesized using a reverse transcription system (Promega, Madison, WI, USA). For semi-quantitative RT-PCR, target genes were amplified using Taq DNA polymerase (Promega) according to the manufacturer's protocol. PCR products were electrophoresed on 2% agarose gels with ethidium bromide and visualized under UV transillumination. Real-time RT-PCR was performed using Thunderbird SYBR qPCR Mix (Toyobo, Osaka, Japan) on a StepOnePlus real-time PCR system (Thermo Fisher Scientific). Gene expression data were analyzed quantitatively using the comparative cycle time (ΔΔCT) method. The primers used are listed in Table S3. *Glyceraldehyde-3-Phosphate Dehydrogenase* (*GAPDH*) expression was used as an internal control.

Immunofluorescence and Immunocytochemistry

Cells were fixed with 10% neutral buffered formalin for 15 min and permeabilized in 0.2% Triton X-100 for 10 min. For immunofluorescence staining, cells were then blocked in 5% bovine serum albumin (BSA; Wako) for 30 min and incubated overnight at 4 °C with primary antibodies against Nanog (Cat# 8822; Cell Signaling, Danvers, MA, USA) or SSEA-1 (Cat# bs-1702R; Bioss, Woburn, MA, USA). The samples were then incubated with Alexa Fluor 488-conjugated secondary antibody (Abcam, Cambridge, MA, USA) for 60 min at room temperature. A fluorescence microscope (Zeiss AxioVert A1, Jena, Germany) was used to observe the staining.

For immunocytochemistry, following fixation and permeabilization as mentioned above, cells were treated with 0.3% H_2O_2 for 30 min and then blocked with 5% BSA for another 30 min. The cells were then incubated with primary antibodies against keratin 14 (Cat# MA5-11599; Thermo Fisher Scientific), amelogenin (AMGN; Cat# sc-33109; Santa Cruz Biotechnology, Santa Cruz, CA, USA), or ameloblastin (AMBN; Cat# sc-50534; Santa Cruz Biotechnology) at 4 °C overnight. The samples

were then incubated with HRP-labeled specific secondary antibodies for 60 min at room temperature. The staining was visualized using a diaminobenzidine (DAB) kit (Roche Diagnostics, Mannheim, Germany). The negative control was cells incubated with 5% BSA instead of the primary antibody.

Western blotting

Cells were lysed using Blue Loading Buffer Pack (Cell Signaling) followed by SDS-polyacrylamide gel electrophoresis of the lysates. The separated proteins were then transferred onto a polyvinylidene difluoride membrane (Bio-Rad, Hercules, CA, USA). After blocking with 5% non-fat milk for 60 min, the membrane was incubated with primary antibodies against keratin 14 (Cat# MA5-11599; Thermo Fisher Scientific), AMGN (Cat# sc-33109; Santa Cruz Biotechnology), AMBN (Cat# sc-50534; Santa Cruz Biotechnology), or β-actin (Cat# 3700; Cell Signaling) at 4 °C overnight. Next, the membranes were incubated with the corresponding HRP-labeled secondary antibodies for 60 min at room temperature. Immunoblot signals were detected using an Immunostar Zeta kit (Wako).

Whole-Transcript Expression Arrays

Total RNA was extracted and purified as described above. RNA purity and integrity were assessed using an Agilent 2100 Bioanalyzer (Agilent Technologies, Santa Clara, CA, USA). Fragmented and labeled cDNA samples were prepared from 250 ng of total RNA using the GeneChip WT Plus Reagent Kit (Thermo Fisher Scientific) according to the manufacturer's protocol. Then, approximately 2.3 μg of fragmented and labeled cDNA was hybridized at 45 °C for 16 h (60 rpm) using GeneChip Hybridization Oven 645 (Thermo Fisher Scientific). Hybridized arrays were washed with a GeneChip Fluidics Station 450 (Thermo Fisher Scientific) and scanned using a GeneChip Scanner 3000 7G (Thermo Fisher Scientific). Raw data were normalized using the robust multi-average (RMA) method and analyzed using Expression Console Software (version 1.1; Thermo Fisher Scientific). Genes with an expression ratio ≥ 2 or ≤ 0.5 compared to the control [Dox(-)] group were defined as upregulated or downregulated, respectively. Functional annotation was performed using Gene Ontology (GO) terms, and GO enrichment analysis was conducted using Metascape (https://metascape.org/) [3].

WST-1 Assay

WST-1 solution (Roche Diagnostics, Mannheim, Germany) was diluted 1:10 in the induction medium at the indicated stage. Absorption was examined at 450 nm by an iMark microplate reader (Bio-Rad, Richmond, CA, USA) after 60 min incubation with the cells.

Scratch Assay

A linear wound was created by a p200 pipet tip followed by washing with PBS twice.

Induction medium of stage 3 containing 1 μg/ml mitomycin C was refreshed, with maintaining Dox treatment. Images were examined at 0 and 48 h after scraping. Cell migration was analyzed by determining the migration area between the cells on either side of the scratch using Image J 1.52a software (Media Cybernetics, Bethesda, MD, USA). The relative migration rate of Dox plus group to the Dox minus group was presented.

Supplemental Figures and Tables

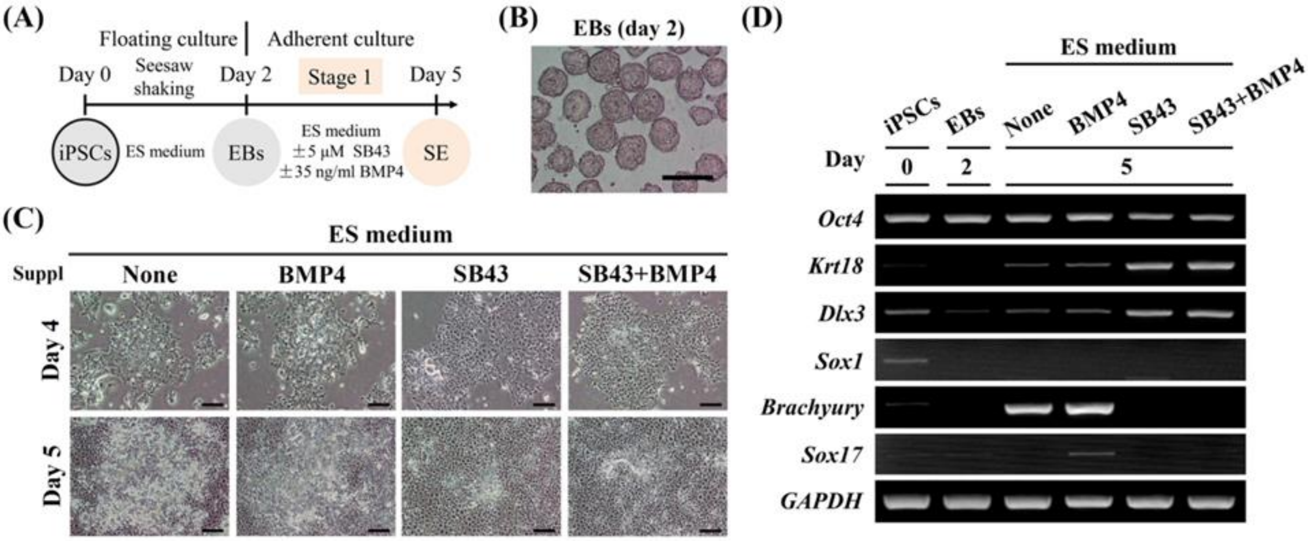

Figure S1. Surface ectoderm (SE) induction (stage 1) from Amelx-iPSCs by inhibition of Nodal signaling. (**A**) Diagram of SE induction from Amelx-iPSCs. After embryoid body (EB) formation by seesaw shaking, the EBs were seeded onto gelatin-coated plates for attachment and incubated in ES medium (iPSC maintenance medium) supplemented with or without SB431542 (SB43; Nodal signaling inhibitor) or BMP4 treatment. (**B**) EB morphology after seesaw shaking for 2 days. Scale bar: 200 μm. (**C**) Cell morphology after stage 1 induction (on days 4 and 5). Scale bars: 200 μm. (**D**) Evaluation of stemness (*Oct4*) and three germ layer markers (SE: *Krt18*, non-neural ectoderm: *Dlx3*, neural ectoderm: *Sox1*, mesoderm: *Brachyury*, endoderm: *Sox17*) by semi-quantitative RT-PCR after stage 1 induction (on day 5).

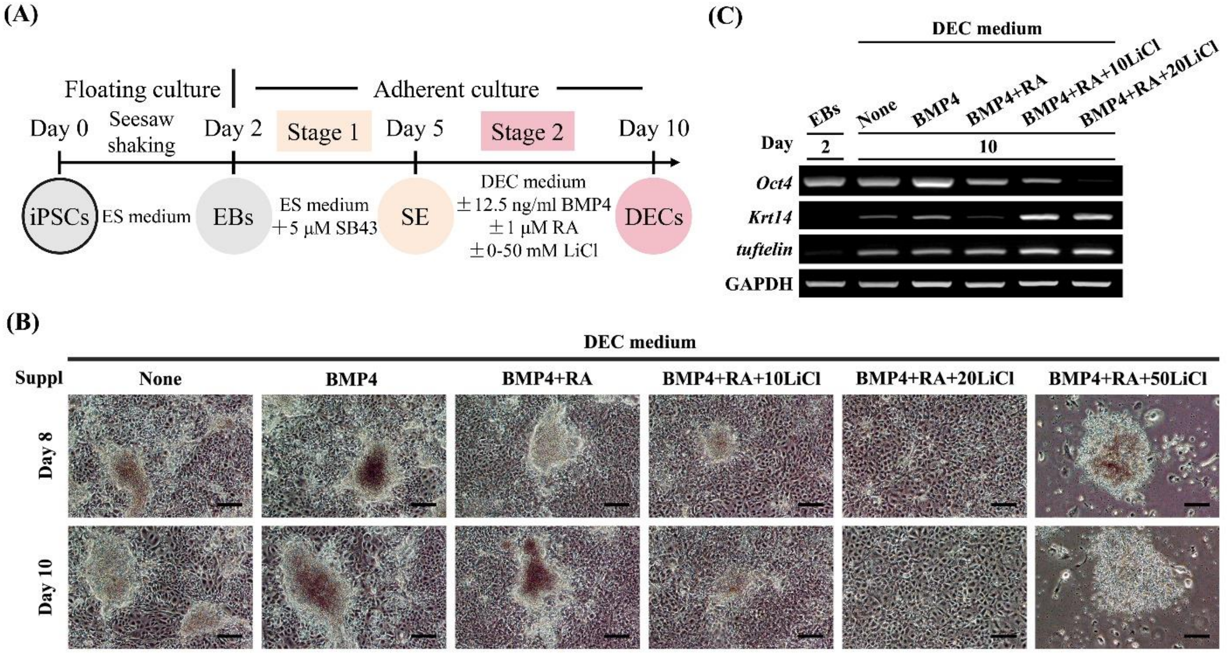

Figure S2. Combination of LiCl, retinoic acid (RA), and BMP4 promoted dental epithelial cell (DEC) induction. (**A**) Diagram of DEC induction of Amelx-iPSCs. After surface ectoderm (SE) induction using SB43, the cells were incubated in DEC medium supplemented with different combinations of BMP4, RA and LiCl (0-50 mM) for DEC induction (stage 2). (**B**) Cell morphology at days 8 and 10 during stage 2 induction. Scale bars: 200 μm. (**C**) Gene expression of stemness (*Oct4*) and DEC (*Krt14* and *tuftelin*) markers determined by semi-quantitative RT-PCR analysis after stage 2 (on day 10).

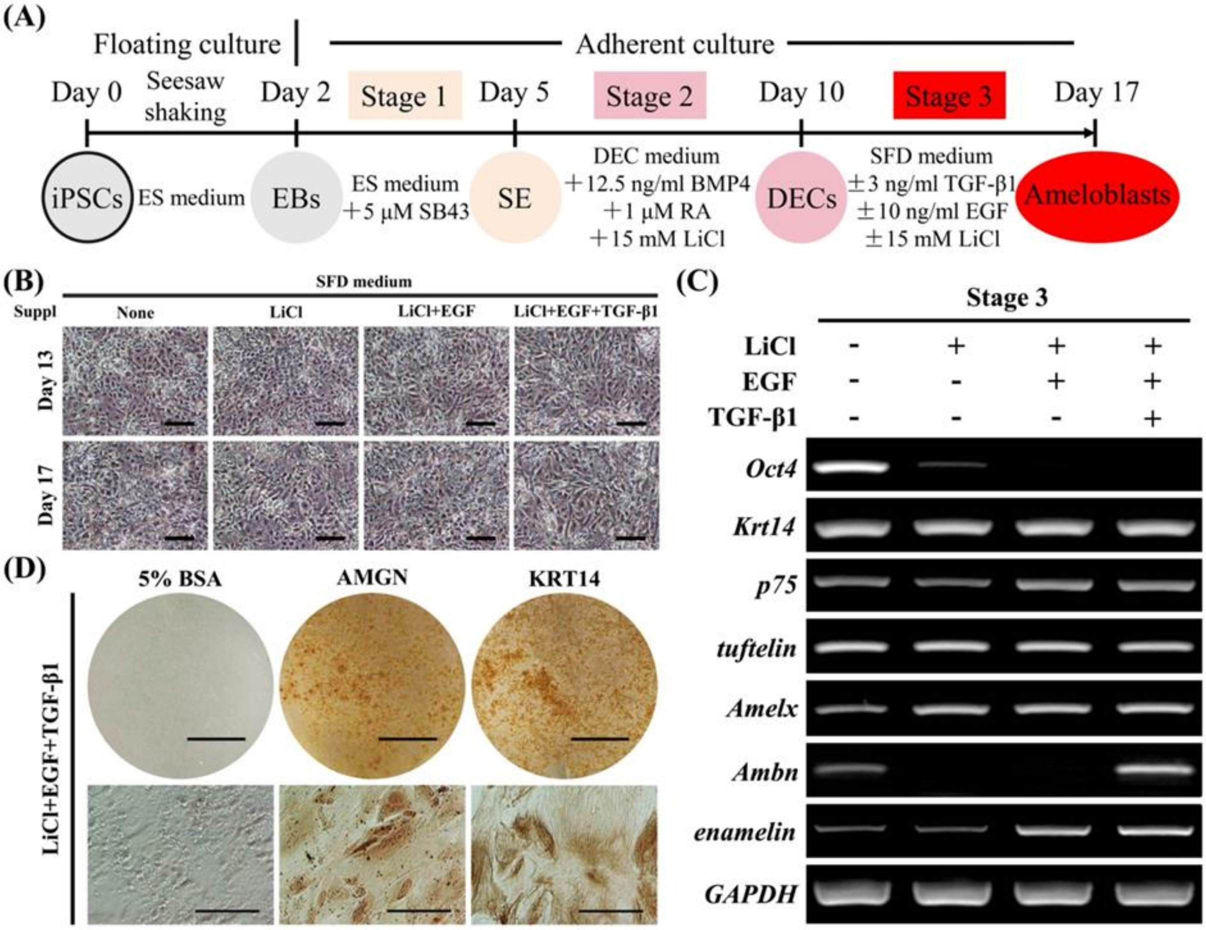

Figure S3. LiCl, EGF, and TGF-β1 cooperated to induce ameloblast lineage differentiation**.** (**A**) Diagram of ameloblast induction from Amelx-iPSCs. Following stage 2 induction using 15 mM LiCl, cells were treated with SF2 differentiation (SFD) medium supplemented with different combinations of 15 mM LiCl, EGF and TGF-β1. (**B**) Cell morphology at days 13 and 17 during stage 3 induction. Scale bars: 200 μm. (**C**) Gene expression of stemness (*Oct4*), DEC (*Krt14*, *p75*, and *tuftelin*) and ameloblast (*Amelx*, *Ambn*, and *enamelin*) markers as determined by semi-quantitative RT-PCR analysis after stage 3 (on day 17). (**D**) Immunochemistry for KRT14 and AMGN on day 17 in Amelx-iPSCs treated with LiCl (15 mM), EGF, and TGF-β1 at stage 3. Scale bars: 1 cm and 100 μm for upper and lower panels, respectively.

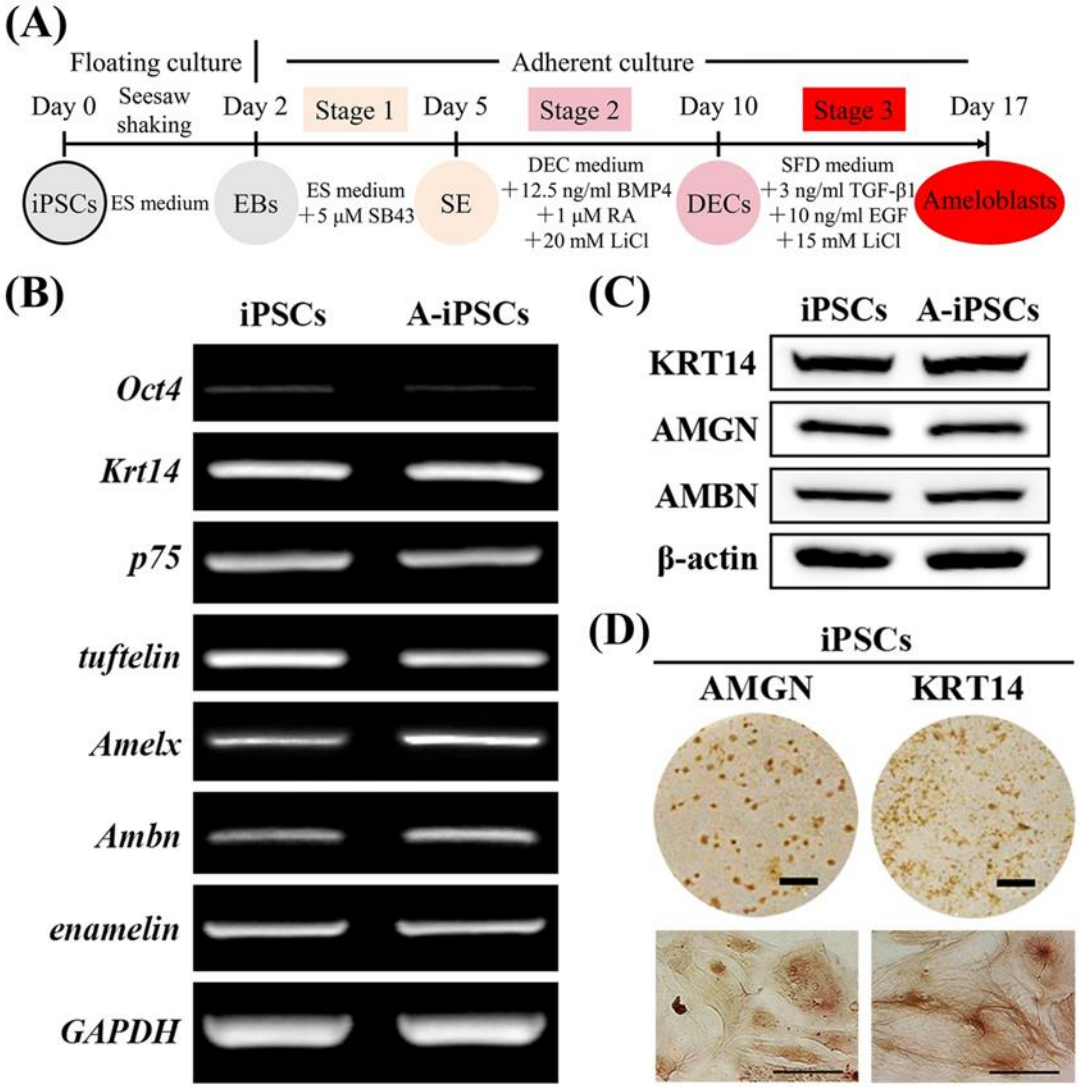

Figure S4. Application of the established stepwise induction protocol to the original mouse iPSCs. (**A**) Diagram of the established stepwise induction protocol. Following stage 2 induction using 20 mM LiCl, cells were treated with SF2-differentiation (SFD) medium with 15 mM LiCl, EGF, and TGF-β1. (**B**) Gene expression of stemness (*Oct4*), DEC (*Krt14*, *p75*, and *tuftelin*) and ameloblast (*Amelx*, *Ambn*, and *enamelin*) markers in original iPSC-derived and Amelx-iPSC (A-iPSC)-derived ameloblasts as determined by semi-quantitative RT-PCR analysis. (**C**) Expression of KRT14, AMGN, and AMBN in original iPSC-derived and A-iPSC-derived ameloblasts as determined by western blotting. β-actin was used as an internal control. (**D**) Immunochemistry for KRT14 and AMGN in the original iPSC-derived ameloblasts. Scale bars: 2 mm and 100 μm for upper and lower panels, respectively.

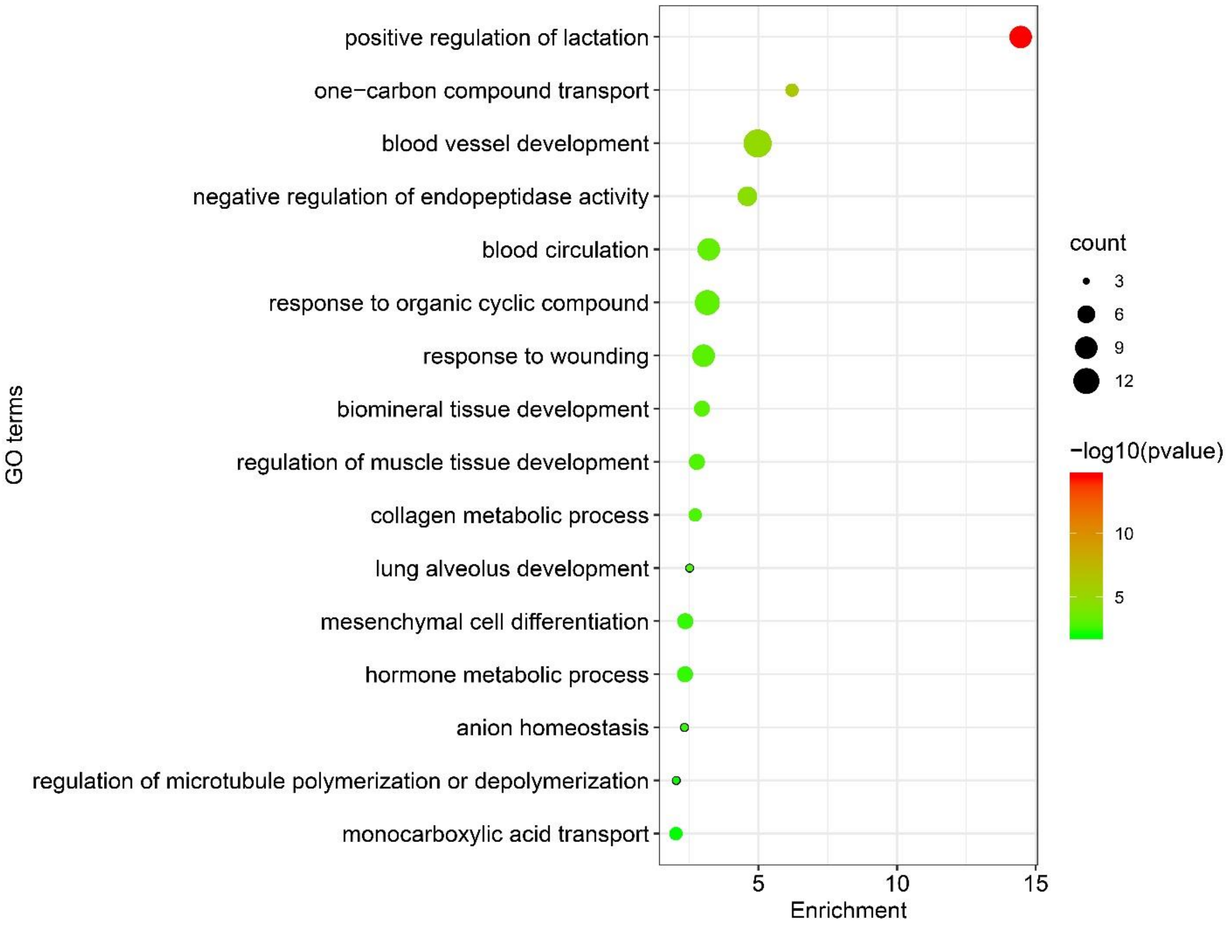

Figure S5. Gene ontology (GO) terms for the downregulated DEGs in the microarray analysis.

Table S1. GO enrichment analysis of upregulated genes.

GO Biological Processes	Genes
Cell-cell adhesion via plasma-membrane adhesion molecules	*Alcam*, ***Cldn3***, ***Nectin3***, *Amigo2*, *Igsf9b*
Regulation of epithelial cell proliferation	*Dlk1*, *Gpc3*, *Six4*, ***Tgm1***, *Dll4*, ***Tacstd2***
Regulation of epithelial cell migration	***Itga2***, *Mmp9*, *Dll4*, ***Tacstd2***
Epithelial cell differentiation	***Cldn3***, *Mmp9*, *Ppl*, ***Tgm1***, *Upk2*, *Scel*
Negative regulation of cell migration	***Cldn3***, *Gnrh1*, *Dll4*, ***Tacstd2***
Negative regulation of cell proliferation	***Cldn3***, *Dlk1*, *Gnrh1*, *Gpc3*, *Mmp9*, *Dll4*
Cell-substrate adhesion	***Itga2***, ***Tacstd2***, *Bcam*, *Muc4*

Bold genes: Upregulation was confirmed by real-time PCR analysis shown in Fig 6D (see main text for details).

Table S2. GO enrichment analysis of downregulated genes.

GO Biological Processes	Genes
Positive regulation of lactation	*Prl3d1*, *Prl2c2*, *Prl2c3*, *Prl7d1*, *Prl2a1*, *Prl2c5*, *Prl3d2*, *Prl3d3*, *Prl2c1*
One-carbon compound transport	*Aqp1*, *Aqp5*, *Car12*, *Slc14a1*

Blood vessel development	*Adm, Aqp1, Cdh5, Col1a1, Col1a2, Dcn, Anpep, Lox, Prl2c2, Prl7d1, Ptprb, Stra6, Serpinb7, Adm2*
Negative regulation of endopeptidase activity	*Aqp1, Serpinb2, Serpinb9c, Serpinb9e, Serpinb9d, Serpinb9g, Serpinb7*
Blood circulation	*Ada, Adm, Cdh5, Col1a2, Htr2b, Anpep, Map2k6, Npy, Adm2*
Response to organic cyclic compound	*Ada, Aqp1, Gsta2, Htr2b, Id3, Igfbp5, Lox, Ncam1, Pappa, Ptgfr, Ifnk*
Response to wounding	*Adm, Aqp1, Col1a1, Lox, Serpinb2, Thbd, Ccn4, Mustn1, Odam*
Biomineral tissue development	*Col1a1, Col1a2, Lox, Srgn, Odam*
Regulation of muscle tissue development	*Igfbp5, Lox, Ncam1, Ccn4, Boc*
Collagen metabolic process	*Col1a1, Col1a2, Rcn3, Serpinb7*
Lung alveolus development	*Ada, Igfbp5, Stra6*
Mesenchymal cell differentiation	*Col1a1, Erg, Htr2b, Frzb, Zeb2*
Hormone metabolic process	*Adm, Crabp2, Cyp11a1, Slc5a7, Slc16a10*
Anion homeostasis	*Slc34a2, Rcn3, Car12*
Regulation of microtubule polymerization or depolymerization	*Cdh5, Stmn2, Stmn4*
Monocarboxylic acid transport	*Fabp4, Slc6a12, Stra6, Map2k6*

Table S3. Primers used for semi-quantitative RT-PCR and real-time RT-PCR.

Gene	Forward primer	Reverse primer	Size (bp)
Mouse primers for semi-quantitative RT-PCR			
Amelx	CAGCAACCAATGATGCCAGTTCCT	ACTTCTTCCCGCTTGGTCTTGTCT	293
Oct4	TCTTTCCACCAGGCCCCCGGCTC	TGCGGGCGGACATGGGGAGATCC	224
Sox2	TAGAGCTAGACTCCGGGCGATGA	TTGCCTTAAACAAGACCACGAAA	297
Nanog	AGGGTCTGCTACTGAGATGCTCTG	CAACCACTGGTTTTTCTGCCACCG	364
keratin18	AGATCGACAATGCCCGCCTT	TGCAGAAGGACCCCATTGAGC	574
Dlx3	TCTGGTTCCAGAACCGCCGCT	TCAGTACACAGCCCCAGGGTTA	341
Sox1	CGGATCTCTGGTCAAGTCGG	GGGACCTCGGTACAAAGTCG	340
Brachyury	CCAGCTCTAAGGAACCACCG	TGTCCACGAGGCTATGAGGA	450
Sox17	TCTGCACAACGCAGAGCTAA	GCATAGTCCGAGACTGGAGC	504
p63	GGAAAACAATGCCCAGACTC	GTGGAATACGTCCAGGTGGC	294
keratin 14	ACCAAAGGCCGTTACTGCAT	GAGGAGAATTGAGAGGATGAGGA	233
p75	ATGGAAGGGGACCGAGATGA	CCGGTGGGGTCCTTCTACTA	571
tuftelin	TCAGCCGTTATCAGCGAGAAG	AGTCAGCGTTCTTGATCCGAA	198
Ambn	TCACCCCTGAATTAGCAGAAGT	GCTCTTGGAAACGCCATGC	174
enamelin	GGAGGAGATCGGAGGGATGT	GGAGAGAAAAAGGCTTAGGGGT	578
GAPDH	CACCATGGAGAAGGCCGGGG	GACGGACACATTGGGGGTAG	418
Mouse primers for real-time RT-PCR			
Amelx	CCCCAGTCACCTCTGCATC	GCTGCATGGAGAACAGTGG	73
Ambn	ATGAAGGGCCTGATCCTGTTC	GTCTCATTGTCTCAAGGCTCAAA	130
Oct4	AGAGGATCACCTTGGGGTACA	CGAAGCGACAGATGGTGGTC	96
tuftelin	TCAGCCGTTATCAGCGAGAAG	AGTCAGCGTTCTTGATCCGAA	198
keratin 14	ACCAAAGGCCGTTACTGCAT	GAGGAGAATTGAGAGGATGAGGA	233
p75	CTAGGGGTGTCCTTTGGAGGT	CAGGGTTCACACACGGTCT	140
Cldn3	ACCAACTGCGTACAAGACGAG	CAGAGCCGCCAACAGGAAA	78
Itga2	TGTCTGGCGTATAATGTTGGC	CTTGTGGGTTCGTAAGCTGCT	100
Nectin3	AAAGGGCCGGATTCTTTAATTGA	TGGCTGACAATCGTTGCTGTT	164
Tacstd2	GTGTGCTGGTGCGTAAACTC	TGGCGCAACTCAATGAGGATG	110
Tgm1	AGACCAGCAGTGGCATCTTC	GCCGCTGCCAGTATACCTTA	132
GAPDH	TGCACCACCAACTGCTTAG	GGATGCAGGGATGATGTTC	177
Rat primers for real-time RT-PCR			
Amelx	CCCCAGTCACCTCTGCATC	GCTGCATGGAGAACAGTGG	73
Cldn3	CGTACAAGATGAGACGGCCA	GACACCGGCACTAAGGTGAG	90
Itga2	ATGGTTCAGGCCACATCCGAG	CCCGCCAGACTCCGGTAA	108
Nectin3	CGGCAAAGCACAACTTTCCT	ACAGAGCCGGGAGAAGAGAA	118

Tacstd2	GTGTGCTGGTGCGTAAACTC	TGGCGCAACTCAATGAGGATG	110
Tgm1	AGACCAGCAGTGGCATCTTC	GCCGCTGCCAGTATACCTTA	132
GAPDH	TTCACCACCATGGAGAAGGC	CTCGTGGTTCACACCCATCA	111

References

1. Egusa, H.; Okita, K.; Kayashima, H.; Yu, G.; Fukuyasu, S.; Saeki, M.; Matsumoto, T.; Yamanaka, S.; Yatani, H. Gingival Fibroblasts as a Promising Source of Induced Pluripotent Stem Cells. *PLOS ONE* **2010**, *5*, e12743, doi:10.1371/journal.pone.0012743.
2. Kim, S.-I.; Oceguera-Yanez, F.; Sakurai, C.; Nakagawa, M.; Yamanaka, S.; Woltjen, K. Inducible Transgene Expression in Human iPS Cells Using Versatile All-in-One piggyBac Transposons. *Methods in Molecular Biology* **2015**, *1357*, 111–131, doi:10.1007/7651_2015_251.
3. Zhou, Y.; Zhou, B.; Pache, L.; Chang, M.; Khodabakhshi, A.H.; Tanaseichuk, O.; Benner, C.; Chanda, S.K. Metascape provides a biologist-oriented resource for the analysis of systems-level datasets. *Nat. Commun.* **2019**, *10*, 1–10, doi:10.1038/s41467-019-09234-6.
